# *Theileria, Babesia*, and *Anaplasma* detected by PCR in ruminant herds at Bié Province, Angola

**DOI:** 10.1051/parasite/2012194417

**Published:** 2012-11-15

**Authors:** M. Kubelová, J. Mazancová, P. Široký

**Affiliations:** 1 Department of Biology and Wildlife Diseases, Faculty of Veterinary Hygiene and Ecology, University of Veterinary and Pharmaceutical Sciences Brno Palackého 1-3 612 42 Brno Czech Republic; 2 Department of Sustainable Technologies, Institute of Tropics and Subtropics, Czech University of Life Sciences Prague Kamýcká 129 165 21 Praha 6 Czech Republic; 3 CEITEC-Central European Institute of Technology, University of Veterinary and Pharmaceutical Sciences Brno Palackého 1-3 612 42 Brno Czech Republic

**Keywords:** tick-borne disease, epidemiology, prevention, diagnostics, blood parasite, Africa, maladies transmises par les tiques, épidémiologie, prévention, diagnostic, parasite du sang, Afrique

## Abstract

Distribution of *Anaplasma* spp., *Babesia* spp., *Theileria* spp., and *Ehrlichia ruminantium*, was for the first time studied in Bié Province, central Angola. We examined 76 blood samples of cattle originated from seven farms, and 13 blood samples of goats from two farms employing molecular genetic tools (PCR). Most prevalent was *A. ovis*-infection in goats (100%) and *A. marginale*-infection in cattle (38% of examined animals, and six out of seven farms). *B. bigemina*-infection was detected in only one specimen at Andulo, whereas *B. bovis* was not detected in Bié. We did not detected *T. parva*, the causative agent of serious diseases in cattle; nevertheless, infection by *T. velifera* was quite frequent (14% of examined animals, and five out of seven farms). Causative agent of heartwater disease – *E. ruminantium*, was not detected. Taking into account short-term perspective of PCR methods in monitoring of epidemiological status in herds, the number of infected animals and distribution of detected pathogens should not be ignored.

## Abstract (fr)

La distribution d’*Anaplasma* spp., *Babesia* spp., *Theileria* spp. et *Ehrlichia ruminantium* a été pour la première fois étudiée dans la province de Bié, au centre de l’Angola. Nous avons examiné par PCR 76 échantillons de sang de bétail provenant de sept fermes et 13 échantillons de sang de chèvres de deux fermes. L’infection par *A. ovis* a été observée chez 100 % des chèvres, celle par *A*. *marginale* chez 38 % des animaux du bétail de six fermes sur sept, celle par *B. bigemina* chez seulement un spécimen à Andulo, tandis que *B. bovis* n’a pas été détecté dans cette province. Nous n’avons pas détecté *T. parva*, l’agent causal de maladies graves du bétail; néanmoins, l’infection par *T. velifera* était assez fréquente (14 % d’animaux examinés de cinq fermes sur sept). *E. ruminantium* n’a pas été détecté. Le recours à la PCR pour le suivi du statut épidémiologique des troupeaux (nombre d’animaux infectés et distribution des agents pathogènes) ne devrait pas être ignoré à court terme.

## Introduction

The tick-borne diseases are still closely related to huge economic losses particularly in developing countries (Uilenberg, 1995). Babesiosis (Tick fever), theilerioses (East Coast fever, January disease, and Corridor disease), ehrlichiosis (Heartwater disease), and anaplasmosis belong to the most important tick-transmitted diseases affecting cattle farming in sub-Saharan Africa (*i.a.* Gachohi *et al.*, 2010; Makala *et al.*, 2003; Okuthe & Buyu, 2006). Worldwide distributed bovine babesiosis is caused by infection with intraerythrocytic parasites *Babesia bovis* and *Babesia bigemina* transmitted by *Rhipicephalus* spp. tick (Sonenshine, 1993). Competent tick vectors *Rhipicephalus evertsi* and *Rhipicephalus (Boophilus) decoloratus* were reported as a dominant tick species in livestock also in Angola (Gomes *et al.*, 1994). The massive death associated with intravascular haemolysis should appear in endemic unstable areas where *B. bovis* is more pathogenic than *B. bigemina*. The most important diseases caused by *Theileria* spp. is undoubtedly connected with *Theileria parva* infection. Acute, frequently fatal East Coast fever is transmitted between cattle by *Rhipicephalus appendiculatus* tick, whereas Corridor disease causing almost 100 % morbidity is transmitted from its asymptomatic carrier African buffalo (*Syncerus caffer*) to cattle also by *Rhipicephalus zambeziensis* and *Rhipicephalus duttoni* (Lawrence *et al.*, 2004). Both, bovine anaplasmosis usually associated with *Anaplasma marginale* infection in cattle, and anaplasmosis of small ruminants caused by *Anaplasma ovis* are distributed worldwide with higher prevalence in tropical and subtropical areas (Bowles *et al.*, 2000). An infection by this obligate intracellular parasite results in mild to severe anaemia and icterus without hemoglobinaemia and hemoglobinuria (Kocan *et al.*, 2010). Febrile heartwater disease is endemic in sub-Saharan Africa and in the islands in the Carribean Sea (Provost & Bezuidenhout, 1987). *Ehrlichia ruminantium*, its agent, causes serious lesions of nervous and circulatory system leading to reduction of cardiac and lung function (Van Amstel *et al.*, 1987).

As a consequence of nearly 30 years lasting civil war, the basic information about current epidemiological status of livestock in Angola is lacking. After the war, domestic animals from different parts of Africa were taken to replace and re-form indigenous herds. We suppose that endemic stability in relation to tick-borne diseases is not understood to these days. In the frame of broader developmental project, we were allowed to perform a brief veterinary survey in Bié Province in Central Angola. Presented paper contributes to increase our knowledge on presence and distribution of four major tick-borne pathogens in this country.

## Materials and Methods

### Blood Sampling and Studied Area

**Fig. 1.**
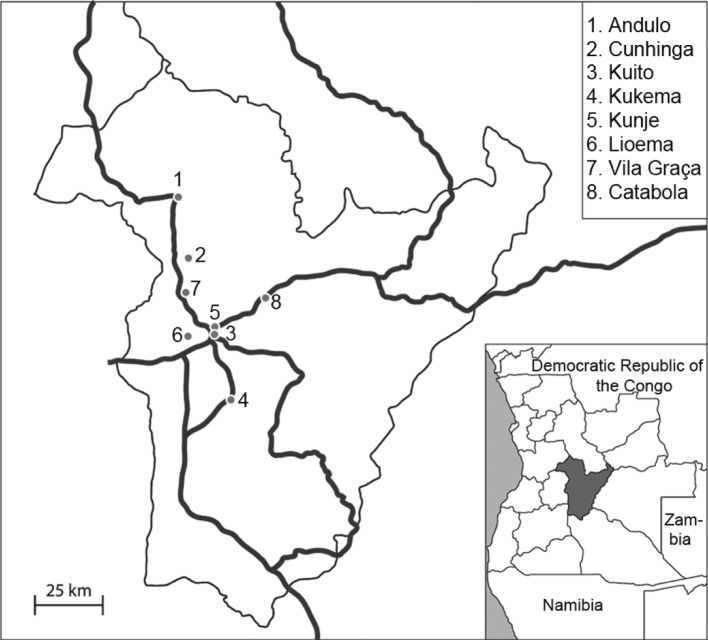
Distribution of sampling sites in Bié Province (dots). Numbers at each locality correspond to those in the Table II. Position of Bié Province in the territory of Angola is evident on small inserted map at right down corner.

Research was carried out in Bié Province in Central Angola during January and February 2009, the months representing there part of the rainy season (http://news.bbc.co.uk/weather/hi/ country_guides; http://www.angola.climatetemp.info/). Altogether 76 cattle blood samples were obtained by puncture of ventral coccygeal vein at seven selected farms operating by mixed farming system – namely in Andulo (n = 6), Cunhinga (n = 4), Kuito (n = 4), Kukema (n = 11), Kunje (n = 11), Lioema (n = 21), and Vila Graça (n = 19) (Fig. 1). All individuals originated from the south of the country (Huíla province) and belonged to local Ovambo breed. Thirteen (13) blood samples were collected from goats living in Catabola (n = 10) and Kuito (n = 3). All selected animals were adults without any symptom of disease. Small amount of collected full blood (0.5 ml) was preserved by surplus of pure 96 % ethanol and transported to laboratory.

### DNA Extraction and PCR Amplification

DNA was isolated from ethanol preserved blood using NucleoSpin Tissue kit (MACHEREY-NAGEL, Germany) following the manufacturer’s protocol, and then stored at - 20 °C until further analysis. Polymerase chain reactions were conducted to amplify specific DNA fragments of *Babesia*/*Theileria* spp., *Ehrlichia ruminantium*, and *Anaplasma marginale*/*ovis*, following modified protocols published by (da Silveira *et al.*, 2011), Mahan *et al.* (1992), and de la Fuente et al. (2001), respectively. All reactions were performed in 25 μl volume – PCR reaction mixture consisted of 12.5 μl Combi PPP Master Mix (Top-Bio s.r.o. Prague, Czech Republic), 10 pmol of each primer (Integrated DNA Technologies, Belgium), 8.5 μl of PCR water (Top-Bio s.r.o. Prague, Czech Republic), and 2 μl of DNA template. In case of nested PCR (detection of *Babesia*/*Theileria* spp.), 1 μl of PCR product from the first round of amplification was used instead of DNA template.

The sequences of the primers and PCR conditions used in the study are presented in Table I. Initial denaturation step at 94 °C lasted five, ten and two minutes for *Babesia*/*Theileria* spp., *E. ruminantium*, and *A. marginale*/ *ovis*, respectively. Final extension step lasted eight minutes at 72° C for *Babesia*/*Theileria* spp., ten minutes at 72 °C for *E. ruminantium*, and seven minutes at 68 °C in case of *A. marginale*/*ovis*. All PCR products were subjected to 1.2 % agarose gel with ethidium bromide and visualized under UV light.

**Table I.** PCR conditions and primers used in the study.

			PCR conditions (°C/s)					
Gene/target	Primers	Sequence 5’– 3’	Denaturation	Annealing	Extension	No. of cycles	Length of PCR product (bp)	References
18S rRNA gene/	RIB19	CGGGATCCAACCTGGTTGATCCTGC	92/60	S4/60	72/120	30	1,700	da Silveira *et al*. (2011)
*Babesia/*	RIB20	CCGAATTCCTTGTTACGACTTCTC						
*Theileria* sp.	BabRumF	ACCTCACCAGGTCCAGACAG	92/60	54/60	72/120	30	420	
	BabRumR	GTACAAAGGGCAGGGACGTA						

*pCS20* gene /E.	AB 128	ACTAGTAGAAATTGCACAATCTAT	94/60	55/60	72/120	4S	279	Mahan *et al*. (1992)
*ruminantium*	AB 129	TGATAACTIGGTGCGGGAAATCCTT						

*msp4* gene/A.	MSP 4S	GGGAGCTCCTATGAATTACAGAGAATTGTTTAC	94/30	60/30	68/60	40	8S1	de la Fuente *et al*. (2001)
*marginale/ovis*	MSP 43	CCCCGGATCCTTAGCTGAACAGGAATCTTGC						

### Dna Sequencing

Representative part of positive PCR products were selected, purified after gel electrophoresis using Gel/ PCR DNA Fragments Extraction Kit (Geneaid Biotech Ltd., Taipei, Taiwan) and sequenced (Macrogen, Amsterdam, Netherlands). Similarity of the obtained sequences was compared with sequences available at GenBank database using BLAST (Basic Local Alignment Search Tool) algorithm (http://blast.ncbi.nlm. nih.gov/) and MEGA program (version 5.05, Tamura *et al.*, 2011).

## Results

All 13 goats’ samples were positive only for the presence of partial sequence of *msp4* gene coding *Anaplasma marginale*/*ovis* surface protein (Table II). Altogether nine out of 13 PCR products were sequenced and all of them revealed 98-100 % similarity to Chinese *A. ovis* isolates deposited in Gen- Bank (HQ456347, HQ456348).

**Table II.** Prevalences of detected tick-transmitted pathogens.

		Number of positive/examined animals (prevalence %)				
	Farm	*Theileria sp.*	*Babesia sp.*	*Ehrlichia ruminantium*	*Anaplasma marginale/ ovis*	*Anaplasma + Theileria*
Cattle	1. Andulo	2/6 (33)	1/6 (16)	0/0 (0)	1/6 (16)*	1/6 (16)
	2. Kunje	3/11 (27)	0/11 (0)	0/11 (0)	6/11 (55)*	2/11 (18)
	3. Vila Graça	3/19 (16)	0/19 (0)	0/19 (0)	10/19 (53)*	2/19 (10)
	4. Kunhinga	0/4 (0)	0/4 (0)	0/4 (0)	1/4 (25)*	0/4 (0)
	5. Kukema	1/11 (9)	0/11 (0)	0/11 (0)	6/11 (55)*	1/11 (9)
	6. Kuito	0/4 (0)	0/4 (0)	0/4 (0)	0/4 (0)*	0/4 (0)
	7. Lioema	2/21 (10)	0/21 (0)	0/21 (0)	5/21 (24)*	0/21 (0)
	Total	11/76 (14)	1/76 (1)	0/76 (0)	29/76 (38)	6/76 (8)

Goats	8. Catabola	0/10 (0)	0/10 (0)	0/10 (0)	10/10 (100)**	0/10 (0)
	6. Kuito	0/3 (0)	0/3 (0)	0/3 (0)	3/3 (100)**	0/3 (0)
	Total	0/13 (0)	0/13 (0)	0/13 (0)	13/13 (100)	0/13 (0)

Numbers of farms correspond to those in Figure 1.

* *A. marginale*;

** *A. ovis*.

We detected gene segments of *A. marginale*, and *Babesia*/*Theileria* spp. in bovine blood samples (Table II). The *msp4* gene of *Anaplasma* spp. was success- fully amplified from 29 bovine samples, 17 of them were sequenced, revealing 99-100 % similarity to an *A. marginale* sequences from Israel (AY786993), Italy (AY702922) and Zimbabwe (AY666011). *A. marginale* was detected at six out of seven farms, with prevalence ranging between 16 % and 55 %. Sequencing of 12 amplified 18S rRNA fragments of *Babesia*/*Theileria* spp. and their following analysis employing MEGA software showed that one sample from Andulo displayed the highest similarity (99 %) with *B. bigemina* isolate originated from Kenya (accession number EF458200). All 11 remaining sequences revealed the highest similarity (99 %) with *Theileria velifera* strain from Tanzania (AF097993). The prevalence of *Theileria* sp. within particular herds ranged between 9 % and 33 %, whereas samples of cattle from Kunhinga (n = 4) and Kuito (n = 4) was PCR negative. In total, we detected six animals being infected by both *A. marginale* and *T. velifera*. We did not find any *E. ruminantium* positive sample. The results are summarized in Table II.

## Discussion

We have diagnosed presence of four important tick-borne haemopathogens among only 89 sampled ruminants from eight farms (Fig. 1). The most prevalent blood parasites in our samples were *A. ovis* (100 %) in goats, and *A. marginale* (38 %) in cattle. Such a high prevalence could be explained by the easy transmission routes, when species of the genus *Anaplasma* can be transmitted by ticks, but mechanical transmission mediated by biting flies and vertical transmission can play a role as well (De Waal, 2000). Biological transmission is effected by more than 20 tick species (Kocan *et al.*, 2004), when at least five competent vector ticks - namely *Argas persicus*, *Rhipicephalus evertsi*, *R. sanguineus*, *R. simus* and *R. (Boophilus) decoloratus* were recorded also from Angola (Walker *et al.*, 2003). We do not expect that all goats in the region are *Anaplasma*-positive; however, the high prevalence of *Anaplasma* in goat herd in sub-Saharan Africa is not a surprise (*e.g.* Bell-Sakyi et al., 2004).

Theilerioses, diseases caused by Apicomplexan parasites of the genus *Theileria*, belong to most important infectious diseases of ruminants in tropics and subtropics (Yusufmia *et al.*, 2010). Among them, infection by different strains of *T. parva* causes the most severe theilerioses of cattle in Africa called East Coast fever (ECF), January disease, and Corridor disease. Whereas *Rhipicephalus appendiculatus*, the vector of ECF, is distributed in eastern Africa, *R. duttoni* and *R. zambeziensis*, both important vectors of Corridor disease, occur naturally also in Angola (Gomes *et al.*, 1994; Walker *et al.*, 2000, 2003). Moreover, earlier reports on clinical cases of Corridor disease in Angola are available (Sonenshine & Mather, 1994). Nevertheless, we did not detect *T. parva* in our samples. It corresponds well with predicting distribution model for theileriosis in sub-Saharan Africa, where Angola remains “untouched” by ECF and by its tick vector *R. appendiculatus* (Olwoch *et al.*, 2008). We found parasites of the genus *Theileria* widely distributed in Bié when sequencing attributes our isolates to *T. velifera*. This *Theileria* species is for long time considered to be non-pathogenic, and thus having low economic importance (Stoltsz, 1989; Bell-Sakyi *et al.*, 2004). Nevertheless, Oura *et al.* (2004) stated that *T. velifera*, *T. mutans*, and *T. taurotragi* can cause in infected cattle ECF-like symptoms. Hence, our finding of *T. velifera* in five herds should not be ignored. Comparing to previous authors reporting mixed infections by different *Theileria* species in Rwanda, Uganda and South Africa (*e.g.* Bazarusanga *et al.*, 2007; Oura *et al.*, 2004; Yusufmia *et al.*, 2010); we did not detect any animal possessing *Theileria* multi-infection.

*B. bovis* and *B. bigemina* are the causative agents of bovine babesiosis in sub-Saharan Africa, where their distribution ranges overlap into large scale (Bock *et al.*, 2004). Among them, *B. bigemina* possess wider distribution, following the distribution of its main vector ticks *R. (Boophilus) decoloratus* and *R. evertsi* (Bock *et al.*, 2004; Walker *et al.*, 2003). We did not find *B. bovis* in our samples collection (data not shown, methodology according to Aboulaila et al., 2010). This situation can be caused by the lack of *R. (Boophilus) microplus* (the main vector of *B. bovis*) in Angola, as published by previous authors (*e.g.* Gomes *et al.*, 1994; Walker *et al.*, 2003). Our results correspond partially with outcomes of survey of 386 bovine sera collected in north-eastern South Africa by Mtshali *et al.* (2004). This team discovered 94 % prevalence of *B. bigemina*, 87 % prevalence of *Anaplasma* sp., and absence of *B. bovis* among studied animals. Despite the fact that we found only 1 % prevalence of *B. bigemina*, due to the time-limited nature of parasitaemia leading to relatively smaller chance to detect *Babesia* by PCR method, this result should not be overlooked.

*Ehrlichia ruminantium*, the causative agent of heartwater disease, was not detected among our samples, despite the fact that *Amblyomma pomposum* and *A. variegatum*, both considered as competent heartwater vectors, were reported from Angola as a frequent tick species infesting cattle (Gomes *et al.*, 1994; Walker & Olwage, 1987). Regarding to the widespread occurrence and dynamics of spreading of heartwater disease intensified with possible vertical transmission (Deem *et al.*, 1996), further study including serological analysis should be carried out to estimate the most imperilled areas by *E. ruminantium* in Angola.

Notably, despite the presence of four above mentioned blood parasites in Central Angola, all cattle and goats were without symptoms of disease. Asymptomatic development of infections could testify their endemic stability in the area. Such a state is defined as relationships between host, vector, and pathogen in environment, when clinical cases or symptoms of disease absent, or occur rarely (Bock *et al.*, 2004). In spite of existence of vaccination programs in Angola, vast majority of farmers cannot support us with information about applied vaccines in their herds due to absence of reliable records. Hence, influence of acquired immune mechanisms (and innate immunity as well) in suppression of symptoms can also play a role. We found mixed infections of *A. marginale* and *Theileria* sp. in six animals. As confirmed by many previous authors, multi-infections in both wild and domestic ruminants of sub-Saharan Africa, are frequent (*e.g.* Awad *et al.*, 2011; Figueroa *et al.*, 1998; Oura *et al.*, 2004; Pfitzer *et al.*, 2011; Simuunza *et al.*, 2011; Yusufmia *et al.*, 2010).

PCR methods give us short-term perspective in monitoring of epidemiological situation calling for employing serological methods. To clarify recent epidemiological threats and to reduce the economic losses in cattle farming in Angola, we suppose as basic the following measures - extensive epidemiological survey employing serology together with molecular genetic methods, monitoring of distribution of tick vectors, availability of vaccination programs, and tracking of animal transports.
